# Increased nitrosative/oxidative stress lowers myocardial protein kinase G activity in heart failure with preserved ejection fraction

**DOI:** 10.1186/2050-6511-14-S1-O2

**Published:** 2013-08-29

**Authors:** Loek van Heerebeek, Nazha Hamdani, Inês Falcão-Pires, Adelino F Leite-Moreira, Mark PV Begieneman, Jean GF Bronzwaer, Jolanda van der Velden, Ger JM Stienen, Gerrit J Laarman, Aernout Somsen, Freek WA Verheugt, Hans WM Niessen, Walter J Paulus

**Affiliations:** 1 Department of Physiology, Institute for Cardiovascular Research, VU Medical Center, Amsterdam, The Netherlands; 2 Department of Cardiology, Onze Lieve Vrouwe Gasthuis, Amsterdam, The Netherlands; 3 Department of Physiology and Cardiothoracic Surgery, University of Porto, Portugal; 4 Department of Pathology, Institute for Cardiovascular Research, VU Medical Center, Amsterdam, The Netherlands; 5 Department of Cardiology, Institute for Cardiovascular Research, VU Medical Center, Amsterdam, The Netherlands; 6 Department of Cardiology, TweeSteden Hospital, Tilburg, The Netherlands

## Background

Prominent features of myocardial remodeling in heart failure with preserved ejection fraction (HFPEF) are high cardiomyocyte resting tension (F_passive_) [1-4] and cardiomyocyte hypertrophy [2]. In experimental models, both reacted favorably to raised protein kinase G (PKG) activity [4,5]. The present study assessed myocardial PKG activity, its downstream effects on cardiomyocyte F_passive_ and cardiomyocyte diameter, and its upstream control by cyclic guanosine monophosphate (cGMP), nitrosative/oxidative stress, and brain natriuretic peptide (BNP). To discern altered control of myocardial remodeling by PKG, HFPEF was compared with aortic stenosis and HF with reduced EF (HFREF).

## Results

Patients with HFPEF (n=36), AS (n=67), and HFREF (n=43) were free of coronary artery disease. More HFPEF patients were obese (P<0.05) or had diabetes mellitus (P<0.05). Left ventricular myocardial biopsies were procured transvascularly in HFPEF and HFREF and perioperatively in aortic stenosis. F_passive_ was measured in cardiomyocytes before and after PKG administration. Myocardial homogenates were used for assessment of PKG activity, cGMP concentration, proBNP-108 expression, and nitrotyrosine expression, a measure of nitrosative/oxidative stress. Additional quantitative immunohistochemical analysis was performed for PKG activity and nitrotyrosine expression. Cardiomyocyte F_passive_ was higher in HFPEF (7.6±0.4 kN/m^2^) than in AS (3.4±0.2 kN/m^2^; *P*<0.001) and in HFREF (5.1±0.2 kN/m^2^; *P*<0.001). In-vitro administration of PKG acutely lowered cardiomyocyte stiffness in all groups with the largest decrement in F_passive_ in DHF patients. PKG activity in myocardial tissue homogenates was significantly lower in HFPEF (5.11±0.62 pmol/min/mg) than in both AS (9.18±0.64 pmol/min/mg; *P*<0.01) and HFREF (11.51±2.0 pmol/min/mg; *P*<0.001). Immunohistochemical determination of myocardial PKG activity by pVASP/VASP ratio provided confirmatory evidence as it was also significantly lower in HFPEF (0.70±0.03) than in both AS (0.84±0.02; *P*<0.001) and HFREF (0.85±0.03; *P*<0.001). Lower PKG activity in HFPEF than in aortic stenosis or HFREF was associated with higher cardiomyocyte F_passive_ (P<0.001) and related to lower cGMP concentration (P<0.001) and higher nitrosative/ oxidative stress (P<0.05). Reduced PKG activity and lower myocardial cGMP concentration in HFPEF did not result from altered myocardial sGC or PDE5A expression, which was similar in all groups nor from unequal BNP expression, which was comparable in HFPEF and AS. The downregulated cGMP-PKG signaling in HFPEF was therefore related to low myocardial nitric oxide bioavailability because of high nitrosative/oxidative stress.

## Conclusion

Low myocardial PKG activity in HFPEF was associated with raised cardiomyocyte F_passive_ and was related to increased myocardial nitrosative/oxidative stress. The latter was probably induced by the high prevalence in HFPEF of metabolic comorbidities. Correction of myocardial PKG activity could be a target for specific HFPEF treatment.
